# Liquid Metal Patterning and Unique Properties for Next‐Generation Soft Electronics

**DOI:** 10.1002/advs.202205795

**Published:** 2023-01-15

**Authors:** Minwoo Kim, Hyungjun Lim, Seung Hwan Ko

**Affiliations:** ^1^ Applied Nano and Thermal Science Lab Department of Mechanical Engineering Seoul National University 1 Gwanak‐ro, Gwanak‐gu Seoul 08826 South Korea; ^2^ Department of Mechanical Engineering Pohang University of Science and Technology 77 Chungam‐ro, Nam‐gu Pohang 37673 South Korea; ^3^ Institute of Advanced Machinery and Design/Institute of Engineering Research Seoul National University 1 Gwanak‐ro, Gwanak‐gu Seoul 08826 South Korea

**Keywords:** liquid metal, liquid metal patterning, soft electronics

## Abstract

Room‐temperature liquid metal (LM)‐based electronics is expected to bring advancements in future soft electronics owing to its conductivity, conformability, stretchability, and biocompatibility. However, various difficulties arise when patterning LM because of its rheological features such as fluidity and surface tension. Numerous attempts are made to overcome these difficulties, resulting in various LM‐patterning methods. An appropriate choice of patterning method based on comprehensive understanding is necessary to fully utilize the unique properties. Therefore, the authors aim to provide thorough knowledge about patterning methods and unique properties for LM‐based future soft electronics. First, essential considerations for LM‐patterning are investigated. Then, LM‐patterning methods—serial‐patterning, parallel‐patterning, intermetallic bond‐assisted patterning, and molding/microfluidic injection—are categorized and investigated. Finally, perspectives on LM‐based soft electronics with unique properties are provided. They include outstanding features of LM such as conformability, biocompatibility, permeability, restorability, and recyclability. Also, they include perspectives on future LM‐based soft electronics in various areas such as radio frequency electronics, soft robots, and heterogeneous catalyst. LM‐based soft devices are expected to permeate the daily lives if patterning methods and the aforementioned features are analyzed and utilized.

## Graphical abstract

Room‐temperature liquid metal (LM)‐based electronics will bring advancements in soft electronics owing to its unique properties. However, patterning LM is difficult because of its rheological features. This resulted in various LM‐patterning methods. A choice of patterning method with understanding is necessary to utilize the properties of LM. Therefore, knowledge about patterning methods and properties for LM‐based soft electronics is provided.

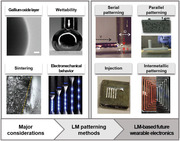

## 1 Introduction

Soft electronics is actively researched owing to an enormous demand for wearable devices, soft robotics, and smart textiles.^[^ ^1^ ^]^ Such electronic invention should be soft and stretchable for stable operation because rigid devices can detach, break, or hinder a user's movement. Because an electrical circuit is indispensable for any electronic device, the first step in fabricating a soft device should focus on developing an electrode that can function stably under mechanical deformation. Presently, liquid metal (LM)**—**LM is defined as gallium‐based room temperature liquid metal, which possesses biocompatibility in this article—appears to be an excellent choice among other conducting materials because it possesses several unique characteristics.

**Table** **1** compares LM with other materials in various aspects. LM has high metallic conductivity compared to other materials, while the high conductivity for nonmetal materials is hard to achieve. LM is the only material that exists in liquid phase at room‐temperature which endows many liquid‐derived properties for soft electronics. Moreover, the cost of LM is relatively chip compared to other materials. LM becomes toxic when sonicated which usually not happen to living creatures, meaning that it is generally nontoxic. To sum it up, LM has liquid‐originated conformability, stretchability with high metallic conductivity, low price and biocompatibility. Therefore, it serves as a suitable electrode material for soft devices, which encounter various deformations. Moreover, surface passivation by gallium oxide helps in adherence to various materials, revealing many substrate candidates. Gallium oxide layer also facilitates fabrication of colloidal LM particle suspension by breaking bulk LM repeatedly. The breakage is generally performed by ultrasonication. By mixing other materials together when ultrasonicated, LM composites with various materials can be easily created. Therefore, when patterning LM, one can choose to use LM in bulk state or colloidal state as well as what composite materials to mix with LM according to target application and fabrication tool.

**Table 1:** Comparison of LM and other conductive materials (EGaIn,^[^ ^103^, ^132^ ^]^ gold,^[^ ^135^ ^]^ silver,^[^ ^136^ ^]^ copper,^[^ ^137^ ^]^ Mxene,^[^ ^138^ ^]^ graphene,^[^ ^139^ ^]^ and PEDOT:PSS^[^ ^140^ ^]^)

	Description	Electrical conductivity [S m^−1^]	Phase at 25 °C	Cost [g per $]	Toxicity	Refs.
EGaIn	Room temperature liquid metal	3.4 × 10^6^	Liquid	0.8 (Rotometals)	Toxic under certain circumstances	[104, 133]
Gold	Noble metal	4.1 × 10^7^	Solid	231 (Sigma‐Aldrich)	Nontoxic	[136]
Silver	Noble metal	6.3 × 10^7^	Solid	13.3 (Sigma‐Aldrich)	Toxic	[137]
Copper	Industrial metal	5.96 × 10^7^	Solid	2.46 (Sigma‐Aldrich)	Toxic	[138]
MXene	2D functional material	Up to 1.1 × 10^6^	Solid	N/A	Toxic under certain circumstances	[139]
Graphene	2D functional material	Up to 2.7 × 10^5^	Solid	N/A	Toxic	[140]
PEDOT:PSS	Conductive polymer	Up to 6.3 × 10^5^	Solid	80 (Sigma‐Aldrich)	Nontoxic	[141]

With LMs unique properties, there are companies that utilize LM to provide their products. For example, Liquid Wire Inc. produces stretchable fluid phase circuitry using liquid metal gel substituting the traditional rigid PCB board. The product can be attached to any textile substrate, and it is washable, stretchable, and fatigue‐free. Similarly, UES Inc. produces ELMNT wearable ink which is a conductive LM based ink. The ink can be painted on elastomeric substrates by various ink‐based patterning methods to create robust, stretchable, and wearable devices.

Although there are even attempts to commercialize LM‐based products, it is in the beginning step. Still, precise LM patterning is hard to be realized due to some hurdles. For instance, the fluidity and spontaneous oxidation of LM serve as an obstacle when handling it. Moreover, its high surface tension limits pattern quality and causes unstable electrical connections. Numerous attempts have been made to overcome the aforementioned drawbacks through precise morphological, mechanical, and chemical modification of LM, its substrate, and their interface. This modification includes forming an alloy with other metals,^[^ ^2^ ^]^ chelating/encapsulating with organic compounds,^[^ ^3^ ^]^ or embedding within polymeric compounds.^[^ ^4^ ^]^ With the aid of such modifications, diverse LM‐patterning methods have been invented. Because each patterning method has its own pros and cons, it has to be chosen judiciously according to a comprehensive understanding of prerequisites, underlying principles, and characteristics of each method. Therefore, a precise investigation of each patterning method is necessary.

In this review, Section 2 outlines key properties of the gallium oxide layer (Section 2.1) and three factors that are necessarily considered for LM patterning (Sections 2.2–2.4): surface energy and thermodynamics of fast forming oxides; mechanical leading to conductive traces; and chemical stability to maintain internal morphology for electromechanical performance.

Then, we categorize LM‐patterning methods into four types (Sections 3.1–3.4): 1) serial patterning, 2) parallel patterning, 3) molding/microfluidic injection, and 4) intermetallic bond‐assisted patterning. Serial patterning refers to ink ejection, ablation, or coalescence of LM done serially.^[^ ^5^ ^]^ It does not require a prepatterned mold or stamp. Parallel patterning refers to methods where LM ink is transferred onto a substrate parallelly. It requires prepatterned structures such as a stencil or stamp. Molding/microfluidic injection requires prepatterned molds and channels for LM to fill in. For molding/microfluidic injection, rheological properties must be considered carefully. Intermetallic bond‐assisted patterning refers to LM forming intermetallic bonding with another prepatterned metal underlayer. Examples of each LM‐patterning method with their details are presented in **Table** **2**.

**Table 2:** Overview of various LM patterning method

Patterning method		Thickness (height) /diameter of LM pattern	Width [µm] of LM pattern	Line spacing of LM pattern	Deformability (strain)	Resistance change under mechanical stress	LM material (+ added composite)	Substrate and interface	Pattern	Application	Refs.
Parallel patterning ‐ additive	Roller‐ball pen	80 µm	200 µm	–	Bending: −180°, −90°, 0°, 90°, 180°	Relatively constant	EGaIn	Paper	Capacitor	–	[43]
	Nozzle‐based method	–	70–80 µm	200 µm (minimum)	–	–	EGaIn	Ecoflex	Electrode, sensor	Curved keyboard, human hand motion sensing device	[51e]
		30–270 µm (diameter)	–	–	–	–	EGaIn	PDMS	LM fiber formation, freestanding wire	3D electronic structure	[58m]
		<2 µm	1.9 µm (minimum)	50 µm	–	–	EGaIn	PET film	(Reconfiguationable electrode) microLED array, antenna	3D electronic structure	[59]
		–	300 µm	–	Stretching: 100%	Stable normalized resistance change (<0.5%, during 1000 cycles)	EGaIn	Dragon Skin 30 (substrate), electroless nickel immersion gold, immersion silver, electroless nickel, organic solderability preservatives, immersion tin (metal electrodes)	soft sensor system	Finger movement monitoring, ground reaction force measurement	[60]
		350 µm	28 mm	–	Stretching: 50%	Relatively constant (during 10000 cycles)	EGaIn	Dragon Skin 30	Sensor, heating element	Haptic device for VR	[73i]
	DoD‐IJP	–	<100 µm	–	Stretching: 60%, bending: 60°, twisting: 180°	Relatively constant	Galinstan	Paper, PDMS	LED circuit electrode	–	[58c]
		–	–	–	–	–	EGaIn	Textiles	Electrode	–	[58e]
Parallel patterning ‐ subtractive	Laser ablation	480 nm	4.5 µm (minimum)	Grating period: 17.5–96 µm	Stretching: >100% (lateral, longitudinal)	Increase linearly to strain (lateral direction)	EGaIn	Cu/Cr (interfacial), PDMS (substrate)	(Grid‐patterned) optochemical response‐abled LM conductor	Transparent air quality monitoring system	[62b]
	Laser‐based PDMS expulsion method	10 µm (minimum film thickness)	20 µm–1 mm	260 µm (minimum)	–	–	EGaIn	PDMS	LED circuit	–	[61b]
	UVLM (UV laser micromachining)	16.3–27.1 µm	50–500 µm	100 µm (minimum)	Stretching: 60%	Close to Pouillet's Law	PDMS, EGaIn, Ag–Fe_2_O_3_ particle	–	Electrode	PPG (photoplethysmogram) recording device	[62a]
Parallel patterning – DC (direct coalescence)	Mechanical DC	–	≈1 µm (coalesced line)	–	–	–	EGaIn (+ grafted with thiol)	Nitrile glove	Strain gauge array	–	[33e]
		10 µm–0.5 mm	7–115 µm (film thickness)	–	Bending: −180° to 180°, folding	Relatively constant to bending, folding	Galinstan	Paper	Recyclable LED circuit electrode	–	[62e]
		–	–	–	Stretching: ≈1200%	Normalized resistance 0.59 (1200% strain)	EGaIn droplet embedded composite (+SIS + PBD)	–	Restorable, reconfigurable, recyclable LED circuit	–	[62f]
		–	500 µm	2.5 mm	Stretching: 50%	Relatively constant	EGaIn droplet embedded composite (+PDMS)	–	Restorable circuit	Self‐healing soft robot	[62g]
	Laser DC	10–20 µm (sintering depth)	200–330 µm	–	Bending: radius of 8–180 mm	Relatively constant	EGaIn	PDMS	LED circuit electrode	–	[31]
		–	90 µm	–	Stretching: 30%	Increase linearly to strain	EGaIn	Sapphire wafer (primary substrate), PDMS (secondary substrate)	Microheater	–	[32c]
Serial patterning ‐ stencil printing/lift‐off method	Stencil printing	<100 µm	–	2.5 mm	Stretching: 400%	Maximum GF 7.16	EGaIn (+SiO_2_, Ni)	Poly(acrylamide‐*co*‐acrylic acid)/ Zr^4+^ (P(AAm‐*co*‐AAc)/Zr^4+^) hydrogel	Strain sensor	Human motion monitoring	[43a]
		20 µm (mask thickness)	85 µm, 2 mm	–	Stretching: 50–375%, bending: curvature 0–5 cm^−1^, twisting: 45°–350°	Stretch: GF 0.86, relatively constant to bending, twisting	EGaIn (+Ni microparticles)	PVA hydrogel	NFC (near‐field communication) antenna	Real‐time human motion monitoring	[63d]
		3 µm	–	–	Stretching: 200%, bending: 15°, 45°, 90°	GF 1.35 (0–100% strain), GF 2.69 (100–200% strain)	Galinstan	TPU (thermoplastic polyurethane)	Strain sensor	Various human movement sensing	[26]
		2 µm	≈100 µm	–	Stretching: 16%	Increase squarely to strain	Ga, In alloy	Skin	IDA electrode array	–	[63]
		1 µm	6 µm (minimum 1:1 line‐and‐space)		–	–	Galinstan	PET, PDMS	Triboelectric nanogenerator (TENG), radio frequency identification (RFID) antenna	–	[67]
		20 µm	500 µm (minimum)	–	–	–	Ga, In alloy	PVC, porous rubber, paper, cloth, tree leaf, plastic film, flask	Electrode on 2D, 3D surface	–	[64b]
		15.51 ± 1.55 µm	Width: 0.8–1.5 mm, resolution: ≈25 µm		Stretching: >1000%	Relatively constant	bGaIn (biphasic GaIn)	VHB tape	Stretch sensor	Sensing normal bending motion of arm	[109]
	Lift‐off method	≈10 µm	20 µm (minimum)	–	Stretching: 60%	Normalized Resistance change of 45% (60% strain)	EGaIn	Si (primary substrate), PDMS (secondary substrate)	Interconnection of transistor based circuit	–	[69a]
		250 nm (minimum)	180 ± 72 nm (minimum)	1 µm (minimum)	Stretching: 30%, bending: radius 5–70 mm, twisting: 0–180°, folding	Relatively constant to bending, twisting	EGaIn	Ti/Au (interfacial), parylene C‐coated Si/SiO_2_ (primary substrate), PDMS (secondary substrate)	Microelectrode array, LED circuit electrode	–	[68]
Serial patterning ‐ TFP (transfer printing)	Stamping	2.4 ± 0.58 µm	15–100 µm	>15 µm	Stretching: 40% (lateral, longitudinal)	–	EGaIn	NTPE SAM‐based PDMS (stamp), PDMS (substrate)	Capacitive strain sensor	–	[73c]
		30–500 µm	20–500 µm	≈40 µm	Stretching: 30%	Relatively constant	Galinstan	cPDMS (stamp), PDMS (substrate)	3D ellipse antenna	–	[17g]
		10–20 µm	Pattern size: <50 µm, resolution: ≈20 µm		–	–	Galinstan	PDMS (stamp, substrate)	Nanogenerator	–	[69b]
	LM filling	<1 µm	2 µm (minimum)	1 µm (minimum)	Stretching: 40%	9.54 Ω (0% strain) 11.27 Ω (40% strain)	EGaIn	PDMS	Coplanar capacitor	–	[73f]
		≈300 nm	2–200 µm	2–100 µm	Bending: radius 13–80 mm, twisting: 0–25°	Relatively constant to bending, twisting	EGaIn	PDMS	LC resonance circuit electrode	3D vertical circuit design	[73d]
		≈1 µm	2 µm (minimum)	>100 µm	Stretching: 30%	Increase (longitudinal stretch), decrease (lateral stretch)	EGaIn	PDMS	LC sensor circuit electrode	Heart rate, blood oxygen monitoring	[73e]
	Dual transfer	–	>100 µm	>150 µm	Stretching: 40% (lateral, longitudinal)	Normalized resistance change <0.4 (during 500 cycle)	Ga, In alloy	PVC (primary substrate), PDMS (secondary substrate)	Electrode	Temperature monitoring system	[18a]
	Evaporation‐based transfer	<50 µm	0.8–10 mm	–	Bending: 0–150°	Relatively constant	EGaIn	PVC (primary substrate), nanocellulose membrane (secondary substrate)	LED circuit electrode	–	[18b]
	Thermal transfer	56 µm (maximum)	1–5 mm	–	Stretching: 300%	Increase during stretch	EGaIn (+Fe microparticles)	PVA (primary substrate), fructose (interfacial), EcoFlex00‐30	Restorable, recyclable circuit	Self‐healing soft robot	[18c]
Molding and microfluidic injection	Molding	≈700 µm (diameter)	–	–	Stretching: 250% (2D pattern)	GF 3.81 ± 0.087 (longitudinal stretch 250% strain) (2D pattern)	Ga	SEBS (mold), EcoFlex00‐30 (encapsulating)	Resistor/capacitor (2D), LED circuit/pressure sensor (3D)	–	[79d]
		–	200 µm (channel width)	–	–	–	EGaIn	PDMS (mold)	LED circuit, antenna, capacitor	–	[80a]
	Microfluidic injection	300 µm (channel height)	200 µm (channel width)	–	Stretching: 250% (*x*, *y* direction), pressure (z direction)	GF 3.93 (*x*‐direction), 3.81(y‐direction)	EGaIn	EcoFlex0030	Pressure sensor	Artificial skin prototype	[42h]
		150 µm	250 µm	–	Twisting: ≈360°, bending: radius 0.15–5 mm	Relatively constant to bending, twisting	Galinstan	PVA	Transient capacitive sensor	Control glove	[98c]
		≈80 µm	75–300 µm	–	Twisting: ≈90°, bending: radius 8.10–20.00 mm	Relatively constant to bending, twisting	EGaIn	PDMS	Circuit electrode/capacitor	Touch keyboard	[80b]
		800 µm (channel height)	1 mm (channel width)	–	–	–	Galinstan	PDMS	RCL filter	–	[18e]
		50 µm (channel height)	1 mm (channel width)	–	–	–	EGaIn	PDMS	Reconfigurable antenna	–	[82i]
	Vacuum filling	50 µm (channel height)	5 µm (minimum channel width)	–	–	–	EGaIn	PDMS	Complex microfluidic patterns	–	[82g]
Intermetallic patterning		–	2–100 µm	50 µm	100%	Change less than 30% ("S" shape wire)	Galinstan	Cu/Au/Cr (interfacial), PDMS (substrate)	Pulse sensor	Human heartbeat measuring device	[82p]
		–	Width: 100–300 µm, resolution: 100 µm		Stretching: 1000%	Relative variation: 4700%	Galinstan	Cu/AgNP(Ag nanoparticle) (interfacial), SEBS (substrate)	LED circuit electrode	–	[14a]
		<3 µm	2.2 µm	15.2 µm (minimum)	Stretching: 50%	GF ≈1 (grid structure)	Ga	Au (interfacial) PDMS (substrate)	Transparent conductor, capacitive sensor	Finger movement monitoring	[82l]
		–	Resolution: 200 µm (maximum)		Stretching: 110%	GF ≈1 (80% strain)	EGaIn	AgNP (interfacial), tattoo film (substrate)	LED circuit electrode	–	[82m]
		70 µm (LM layer)	420 µm	–	Stretching: 200%	Normalized resistance change 40% (200% strain)	Galinstan	AgNP (interfacial), SBS (substrate)	Electrode	Remote controlling drone with hand gesture	[81]
		200 µm (circuit), 40 µm (Galinstan)	4 mm	–	–	–	Galinstan	Cu + CuCl_2_ solution (interfacial), PVC (substrate)	Electrode	–	[82o]
		–	40 µm (minimum)	–	Stretching: <84.5 ± 9.4%	Close to Pouillet's Law (60% strain)	EGaIn	Cu (interfacial), PDMS(substrate)	Accelerometer/temperature sensor circuit electrode	–	[82q]
		50 µm	Resolution: 100 µm		Stretching: 800%	Increase ≈30 times (800% strain)	EGaIn	Cr/Cu (interfacial), SEBS(substrate)	Strain sensor	In vivo heart motion monitoring of frog, rabbit	[83]
		2.5 µm	4 µm (microstructure size)	8 µm (pitch)	Stretching: 80%	Increase during stretch	Ga	Au (interfacial), PDMS (substrate)	Ga film	–	[84]
		<25 µm	25 (microstructure size, minimum)	25–100 µm (microstructure spacing, minimum)	Stretching: 70%	Close to Pouillet's Law (60% strain)	EGaIn	Cu (interfacial), PDMS (substrate)	Ga film	–	[85]

**Figure 1:**
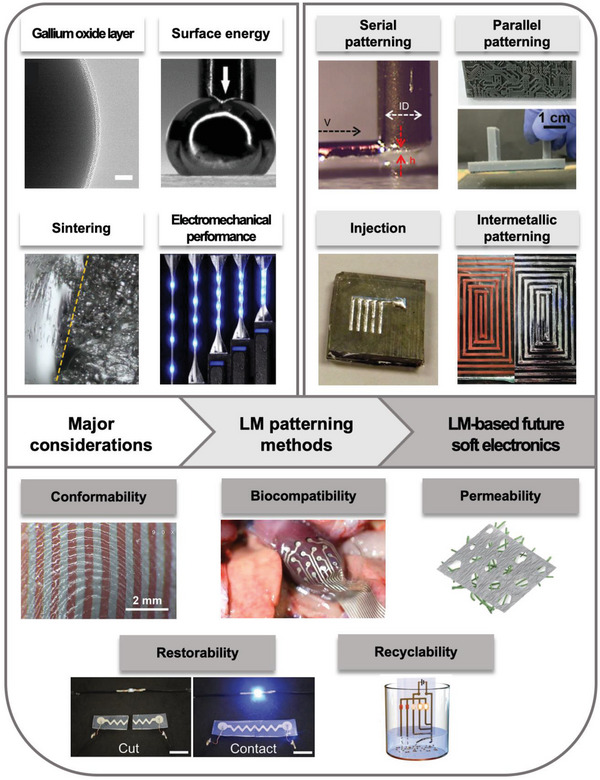
Systematic diagram. Image for “gallium oxide layer”. Reproduced with permission.^[^ ^32a^ ^]^ Copyright 2015, Wiley‐VCH. Image for “surface energy”. Adapted with permission.^[^ ^7c^ ^]^ Copyright 2014, American Chemical Society. Image for “sintering”. Reproduced with permission.^[^ ^36a^ ^]^ Copyright 2021, MDPI. Image for “Electromechanical performance”. Reproduced with permission.^[^ ^115c^ ^]^ Copyright 2021, Springer Nature. Image for “serial patterning”. Reproduced with permission.^[^ ^51e^ ^]^ Copyright 2019, Wiley‐VCH. Image for “parallel patterning”. Reproduced with permission.^[^ ^28^ ^]^ Copyright 2020, Wiley‐VCH. Image for “injection”. Reproduced with permission.^[^ ^81c^ ^]^ Copyright 2017, The Royal Society of Chemistry. Image for “intermetallic patterning”. Reproduced with permission.^[^ ^90^ ^]^ Copyright 2021, Wiley‐VCH. Image for “conformability”. Reproduced with permission.^[^ ^101^ ^]^ Copyright 2021, AAAS. Image for “biocompatibility”. Reproduced with permission.^[^ ^94^ ^]^ Copyright 2022, AAAS. Image for “permeability”. Reproduced with permission.^[^ ^112^ ^]^ Copyright 2021, Wiley‐VCH. Image for “restorability”. Reproduced with permission.^[^ ^67^ ^]^ Copyright 2019, Wiley‐VCH. Image for “recyclability”. Reproduced with permission.^[^ ^120^ ^]^ Copyright 2019, Wiley‐VCH.

Next, we provide unique properties that LM‐based soft electronics possesses, which are conformability, biocompatibility, permeability, restorability, and recyclability (Section 4.1). These properties presented in Section 4.1 can be exceptionally satisfied by utilizing the outstanding features of LM with diverse patterning methods. Also, we provide future perspectives on LM‐based soft electronics in various areas such as radio frequency (RF) electronics, soft robotics, and heterogeneous catalyst. We expect LM‐based soft devices to successfully pervade our daily life if the features presented in this review are fully investigated and exploited. A systematic diagram of the overall contents in this review is presented in **Figure** **1**.

## 2 Major Considerations for Liquid metal (LM) Patterning

### 2.1 Gallium Oxide Layer

Because most LM‐patterning processes are conducted in ambient air conditions where a spontaneous oxide layer forms, considering the oxide layer and its interaction with substrates or surroundings is necessary.^[^ ^6^ ^]^ The presence of oxygen promotes the formation of an oxide layer at the outer surface of the LM.^[^ ^7^ ^]^ An oxide layer with a thickness of a few nanometers is composed of Ga_2_O_3_, Ga_2_O, and relatively low density of In_2_O_3_ due to the high oxidation tendency of gallium compared to indium (and tin, in the case of Galinstan).^[^ ^8^ ^]^

Since LM patterning is closely related to controlling oxide layer and wettability, qualitative measurement for LMs wetting behavior is important. However, conventional static contact angle measurement provides inconsistent value according to the experimental circumstances due to rigid LM oxide's adhering behavior. Therefore, Joshipura et al. suggested measuring advancing and receding contact angle while considering about substrate surface's morphology and chemistry instead of measuring unstable static contact angle.^[^ ^9^ ^]^

Pure Ga and GaIn alloys (such as EGaIn and Galinstan) show high surface tension ≈500–700 mN m^−1^ in low oxygen pressure environment or removal of surface oxide layer with strong acid/base solution.^[^ ^7^, ^10^ ^]^ However, when LM is surrounded by the oxide layer, its interaction with other materials is generally increased, which is mainly due to electrostatic interaction and covalent bonding between the oxide and high‐surface energy substrates such as metallic layers or polar group‐exposed polymers.^[^ ^11^ ^]^ This strong interaction facilitates LM adherence to a substrate or produces a stable colloidal solution by surface grafting.^[^ ^12^ ^]^ For example, Guo et al. applied PMA glue to pattern LM on a paper substrate because PMA undergoes a strong chemical interaction with the LM oxide layer.^[^ ^13^ ^]^ Lin et al. produced stable aqueous LM nanoparticles grafted with poly(1‐octadecene‐alt‐maleic anhydride) (POMA) solution. They determined that the production was possible because hydrolyzed carboxylic acid groups in POMA served as emulsifiers, which interacted with the LM oxide layer.^[^ ^12c^ ^]^

In some LM‐patterning studies, the oxide layer is intentionally removed by treating an acid or a base solution to modify the adherence behavior of LM.^[^ ^7^, ^14^ ^]^ For example, Kim et al. revealed that HCl vapor generated nonwetting Galinstan by altering the oxide layer component from Ga_2_O_3_ to GaCl_3_ and InCl_3_.^[^ ^15^ ^]^ In a subsequent research, Kim et al. demonstrated that HCl treatment of a paper substrate led to less adherence of Galinstan.^[^ ^14a^ ^]^ As demonstrated by previous studies, oxide layer removal generally induces nonwetting behavior for LM. However, an oxide‐removed LM exhibits strong wetting behavior on metals such as Au, Ag, and Cu.^[^ ^16^ ^]^ This behavior is attributed to the intermetallic bonding that occurs when a direct contact between the LM core and other metals occurs. This phenomenon has resulted in a unique patterning method called intermetallic bond‐assisted patterning.

### 2.2 Surface Energy and Thermodynamics of Fast Forming Oxides

There are numerous LM‐patterning methods, and each method requires a different degree of wettability.^[^ ^16^ ^]^ Because a pure LM possesses high surface tension, which generally results in poor wettability, researchers directly utilize this high surface tension with low wettability or, in most cases, reduce the tension for high wettability through various techniques for different circumstances.^[^ ^17^ ^]^ The techniques involve LM ink modification, interface modification, or substrate selection.^[^ ^18^ ^]^

**Figure 2:**
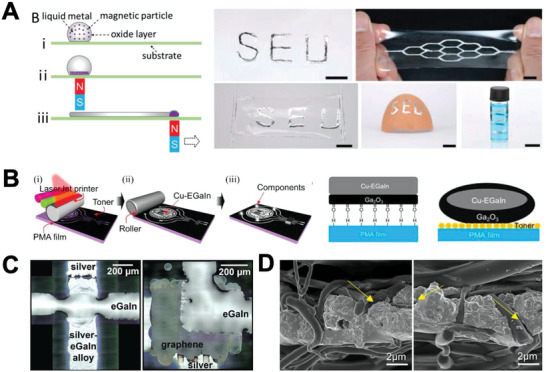
Wettability control. A) Wetting behavior of LM ink with Ni microparticles under magnetic field intervention (left), LM patterns on various substrates (right). Reproduced with permission.^[^ ^22^ ^]^ Copyright 2019, Wiley‐VCH. B) Selective wetting Cu‐EGaIn on toner/PMA substrate (left), difference in LM adherence on PMA and toner (right). Reproduced with permission.^[^ ^25^ ^]^ Copyright 2020, The Royal Society of Chemistry. C) EGaIn/silver with alloy formation (left), EGaIn/graphene/silver with no alloy formation (right). Reproduced with permission.^[^ ^26^ ^]^ Copyright 2017, Wiley‐VCH. D) SEM images of interaction between TPU nanofiber and LM. Adapted with permission.^[^ ^29^ ^]^ Copyright 2021, American Chemical Society.

One representative approach for controlling wettability is to prepare the LM ink by mixing with other materials.^[^ ^20^ ^]^ For instance, Hao et al. reported that LM mixed with Ni particles had a strong interfacial bonding with a hydrogel substrate and led to a noticeable increase in pattern fidelity and stability. The group determined that the LM–Ni composite ink with enhanced wettability facilitated uniform spreading on the substrate, which resulted in strong bonding.^[^ ^21^ ^]^ Similarly, Ma et al. proposed a magnet‐assisted patterning method using LM–Ni composite ink. Ni microparticles in the ink induced LM movement, which followed the trace oriented with the magnet (0.4 T). The group found that a strong adhesion between the LM–Ni composite and paper substrate was created owing to enhanced wettability. Further, they demonstrated that the enhanced wettability between the composite and the substrate remained after removal of the magnet. Such a method was used to realize patterning on paper, PDMS, hydrogel, and an egg shell with curved geometry^[^ ^22^ ^]^ (**Figure** **2**A).

Substrate and ink interface modification has also been widely studied for wettability control.^[^ ^22^ ^]^ For example, Wu et al. conducted ethyl‐2‐cyanoacrylate polymerization at the interface of Galinstan and stretchable polyacrylate substrate. The modified interface led to a strong bonding of Galinstan on the substrate, resulting in a stable electrical connection at a strain over 80%.^[^ ^23^ ^]^ Similarly, Guo et al. reported that an LM–Cu particle composite selectively adhered to bare PMA but not to the toner‐covered region. The group observed that the LM oxide layer formed hydrogen bonding with a smooth and bare PMA film, where the toner‐covered region had a rough surface, which reduced the contact area with the oxide, resulting in poor wettability. They fabricated an e‐skin for wearable electronics by utilizing this selective wetting behavior for patterning circuits^[^ ^24^ ^]^ (Figure 2B). Secor et al. demonstrated that interface modification could prevent distortion of a circuit. The group reported that LM lines were intact when a graphene interfacial layer was placed between a silver wire and LM, while LM lines without graphene were distorted. They explained that the interfacial layer acted as an alloy‐restricting barrier that alleviated the distortion caused by reactive wetting between Ag and LM^[^ ^25^ ^]^ (Figure 2C).

Substrate selection or designing is an alternative way of ink or interface modification to achieve wetting behavior.^[^ ^27^ ^]^ For instance, Park et al. reported that a hydroxyl group‐based hydrogel exhibited strong adhesion with LM because of the interaction between the LM oxide layer and its functional group. Consequently, the LM deformed together with its hydrogel substrate when stretched without delamination. Such spontaneous wetting between LM and hydrogel was named “surface reconciliation” in this research.^[^ ^28^ ^]^ Fiber‐based substrates also possess high wettability for LM owing to increased surface area, with which the LM can interact. For example, Wang et al. reported that an LM‐wetted thermoplastic polyurethane membrane exhibited stable electrical connection with mechanical robustness. The membrane, which was prepared by electrospinning, was composed of numerous fibers. The group explained that the LM and porous fiber membrane created an interpenetrating structure that facilitated reliable connection (Figure 2D).^[^ ^29^ ^]^ Similarly, Ma et al. demonstrated an LM‐coated electrospun SBS fiber mat, where intimate connection between the LM and mat was achieved through repeated stretching and releasing cycles. The group found that the repeated cycles induced continuous breakage and formation of an oxide layer, which resulted in wetting of the mat as well as conductivity gain of the LM.^[^ ^30^ ^]^

### 2.3 Mechanical Leading to Conductive Traces

After LM particle patterning is accomplished, the oxide layer in each individual particle hinders electrical connection, which results in an insulating pattern.^[^ ^31^ ^]^ In such a situation, sintering is necessary to rupture the particle and lead the inner liquid core to flow out to form continuous electrically conducting interconnections. Accordingly, mechanical,^[^ ^32^ ^]^ thermal,^[^ ^33^ ^]^ and chemical sintering^[^ ^34^ ^]^ or other methods^[^ ^3^, ^35^ ^]^ are frequently conducted.

**Figure 3:**
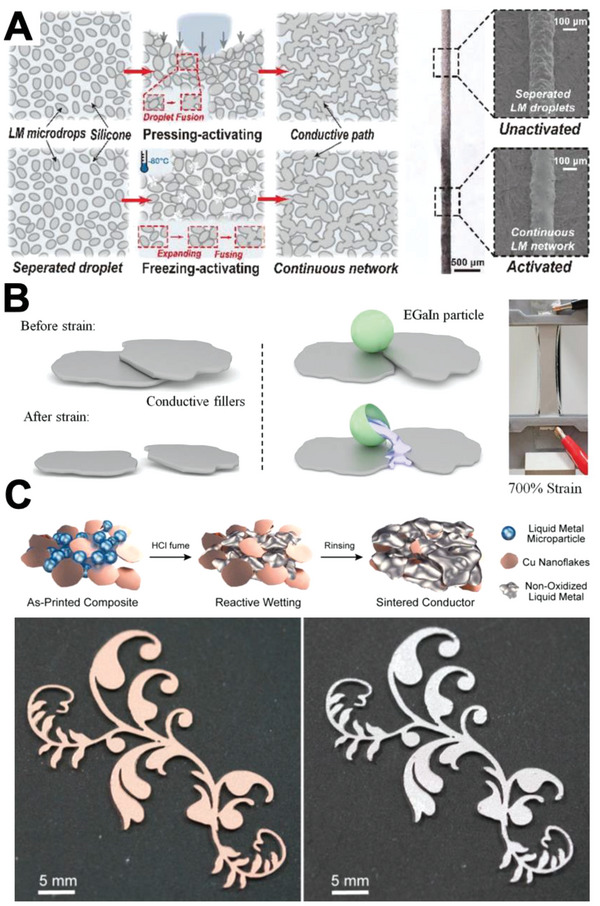
Sintering methods. A) Schematic illustration of pressing and freezing sintering (left), comparison of optical and SEM images between activated/unactivated LM‐silicone line (right). Reproduced with permission.^[^ ^37^ ^]^ Copyright 2019, Wiley‐VCH. B) Illustration of conductor under stretch with/without LM particles (left), conductor under 700% strain (right). Reproduced with permission.^[^ ^32b^ ^]^ Copyright 2018, Wiley‐VCH. C) Schematic illustration of chemical sintering process (top), image of LM/Cu pattern before (bottom left) and after (bottom right) sintering. Adapted with permission.^[^ ^34^ ^]^ Copyright 2020, American Chemical Society.

Mechanical sintering is performed in various ways such as applying shear force, compressive force, tension force, or force induced by thermal expansion.^[^ ^32^, ^36^ ^]^ In order to accomplish mechanical sintering, the mechanical force must break the oxide shell and let the LM core leak to form a connected electrical pathway. For instance, Huang et al. fabricated an LM‐coated polydopamine‐treated polyurethane sponge and conducted sintering by pressing the sponge. The mechanical stress applied by pressing induced LM nanoparticles in the sponge to rupture, or be sintered.^[^ ^32c^ ^]^ Similarly, Zhou et al. fabricated an LM–silicone composite by mixing PDMS with LM particles and curing them. The embedded LM droplets were sintered by pressing the composite. Additionally, the group reported that a freezing process could also create conductive pathways because of expansion, rupture, and coalescence of LM droplets (**Figure** **3**A).^[^ ^37^ ^]^ Wang et al. mixed LM particles with Ag flakes, Ag nanoparticles, and ethylene vinyl acetate to fabricate a superelastic conductor. The group explained that the conductor could achieve superelasticity with high conductivity because the LM particles were smeared, or sintered, between fillers when stretched, serving as a conductive interconnection^[^ ^32b^ ^]^ (Figure 3B).

Mechanical sintering method is frequently utilized in LM‐based reconfigurable circuits. The word “reconfigurable” means erasing a previous circuit and rewriting a new one. For example, Park et al. fabricated an LM‐embedded poly(ethylene glycol) diacrylate (PEGDA) hydrogel composite. Conductive lines sintered by mechanical force could be erased by spraying water over the composite. Water caused swelling of the PEGDA substrate, which resulted in the destruction of the original conductive paths. After the composite dried, the upper portion of the elastomer was reconstructed with unruptured LM particles, which was capable of rewriting. The group successfully modified LM circuits into different configurations through repeated processes of direct writing, erasing, and rewriting.^[^ ^38^ ^]^ Similarly, Tutika et al. reported a rewritable and restorable SIS–PBD (polybutadiene)–LM composite. LM droplets embedded inside the elastomer formed conductive pathways when coalesced. Because SIS was soluble to toluene, the group erased the original LM circuit by exposing the composite to toluene, and a new circuit could be written, realizing a reconfigurable circuit.^[^ ^39^ ^]^

Thermal energy can also induce sintering. Liu et al. demonstrated two ways to achieve sintering by thermal energy—thermal sintering and laser sintering—and compared surface morphologies that were observed on the LM particle film. Both sintering methods induced LM nanoparticles to undergo expansion, anisotropic contraction, and sintering. However, thermal sintering, which was conducted in a heated oven, generated less concentrated heat stress and induced more oxidization in LM particles, which led to the production of nanowire structures. However, laser sintering induced extensive oxide rupture and resulted in a coalesced film due to highly concentrated heat stress in a short period of time. Despite the difference in surface morphology, both methods successfully realized sintering by thermal energy.^[^ ^33d^ ^]^ In addition, Wallace et al. proposed a photonic method utilizing pulsed light from a lamp for sintering an LM nanoparticle pattern that was formed by stencil printing. The group explained that the LM pattern was sintered by thermal energy from the lamp assisted by exothermal combustion of nitrocellulose.^[^ ^35b^ ^]^

Other sintering methods such as evaporation sintering^[^ ^3^, ^40^ ^]^ and chemical sintering^[^ ^41^ ^]^ have also been proposed. For instance, Yuen et al. reported evaporation‐based self‐sintering of LM particles in aqueous 25% EtOH solution. The group proposed that sintering was achieved through capillary force induced by evaporation, which caused coalescing of neighboring LM particles.^[^ ^35a^ ^]^ On the other hand, Li et al. presented chemical sintering by exposing an LM/Cu composite to HCl vapor. The vapor removed the oxide layer of LM particles to induce sintering, or reactive wetting in this case, between the LM and Cu flake, forming a highly deformable and conductive composite^[^ ^34^ ^]^ (Figure 3C).

### 2.4 Chemical Stability to Maintain Internal Morphology for Electromechanical Performance

Electrical response to mechanical strain, or electromechanical behavior, is a major factor for maintaining reliable performance of stretchable and soft devices. The representative parameter of this response, or the gauge factor (GF = (Δ*R*/*R*)/(Δ*L*/*L*), where *R* is resistance, Δ*R* is variation in resistance, and Δ*L*/*L* is mechanical strain), is severely affected by LM ink composition, substrate, interface material, microscale structure, and patterned geometry.^[^ ^9^, ^42^ ^]^ Generally, an electrode with low GF is considered a suitable component for electrical circuits, whereas an electrode with high GF is considered a suitable component for sensors.

**Figure 4:**
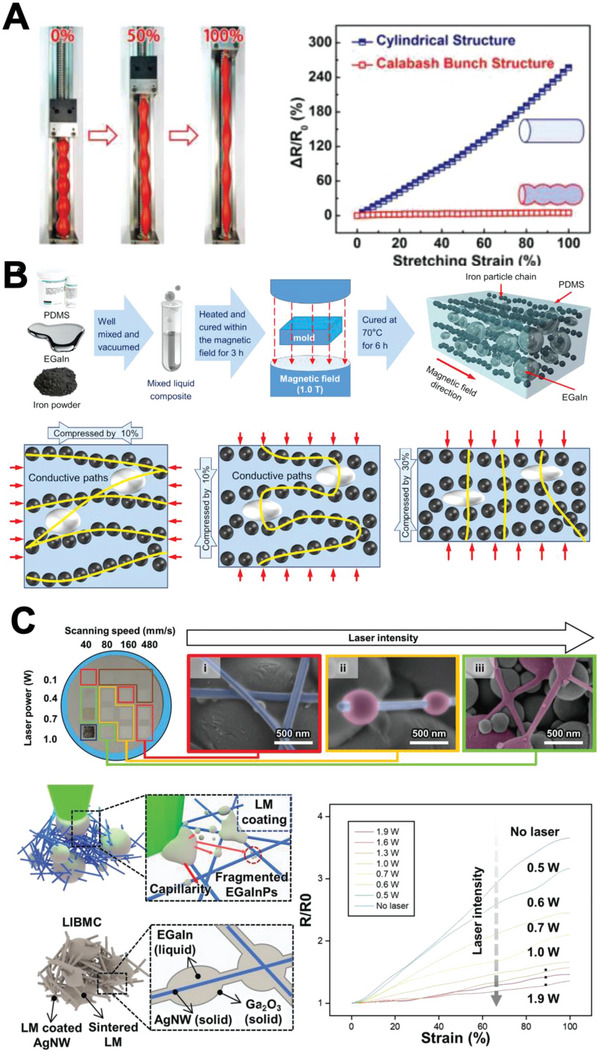
Electromechanical response. A) Calabash Bunch structure under mechanical strain (left), graph of normalized resistance variance to strain for cylindrical/Calabash Bunch structured rubber with Galinstan fillers. Reproduced with permission.^[^ ^43d^ ^]^ Copyright 2018, Wiley‐VCH. B) Illustration of anisotropic liquid metal‐filled magnetorheological elastomer (ALMMRE) and its fabrication process (top), variation of conductive pathways with different compression profiles (bottom). Reproduced with permission.^[^ ^47^ ^]^ Copyright 2020, Elsevier. C) SEM images of EGaIn particle/silver nanowire (AgNW) network with varying laser intensity and laser power (top), illustration of EGaIn particle fragmentation under laser scanning (bottom left), change in resistivity due to strain with varying laser intensity (bottom right). Reproduced with permission.^[^ ^48^ ^]^ Copyright 2022, Wiley‐VCH.

Considerable efforts have been devoted to fabricating a conductor with low GF via various mechanisms.^[^ ^42^ ^]^ For example, Liu et al. proposed that biphasic GaIn, which was composed of solid Ga_2_O_3_ particles, and LM exhibited extreme stretchability over 1000% strain with negligible resistance change. They explained that the negligible change in resistance originated from the decreasing volume of semiconductive solid Ga_2_O_3_ particles in the biphasic layer while being stretched. In addition, they explained that biphasic GaIn could maintain its continuous conductive pathway under extreme strain via the crack‐filling phenomenon of the LM.^[^ ^42^ ^]^ Similarly, Park et al. fabricated a Ni flakes–LM–carboxylated polyurathane composite, which exhibited negligible resistance change under strain. The group explained that a low variation in resistance was achieved because cavities created upon stretching were autonomously filled with LM.^[^ ^42^ ^]^ Yu et al. reported another research on an LM conductor that exhibited low resistance change when stretched. They fabricated the conductor by injecting Galinstan into a PDMS cylinder with a Calabash Bunch structure. They reported that when the entire structure was stretched, the strain was mainly released by the calabash balls with high curvature. This resulted in less effective strain as well as low resistance variation^[^ ^42^ ^]^ (**Figure** **4**A).

Meanwhile, LM electrodes with a dramatic change in conductivity under mechanical stress can be utilized as sensors.^[^ ^44^ ^]^ Moreover, an LM composite sensor exhibits many unique electromechanical properties owing to its solid–liquid biphasic structure. For instance, Wu et al. reported a soft sensor made of an LM–SiO_2_–Ni composite that exhibited a high GF of 7.16 while stretching up to 400%. The group explained that a high GF could be obtained because of SiO_2_ particles, which clustered under stretching and generated strain concentration.^[^ ^45^ ^]^ By the way, a series of studies conducted by Yun et al. demonstrated that PDMS with embedded LM–Fe microparticles presented positive piezoconductivity. The group analyzed that the positive piezoconductivity stemmed from a larger contact area with decreased distance between conductive particles under elastomer deformation. The composite was successfully utilized as a sensor to monitor finger‐bending motion.^[^ ^46^ ^]^ In a later research, Yun et al. aligned Fe particles in a PDMS–LM–Fe composite using a magnetic field. The composite exhibited varying degrees of piezoconductivity according to the direction and intensity of stress. The group analyzed that such an electrical property originated from anisotropic variation in tortuosity of the conductive pathway with aligned Fe. They fabricated an adjustable rheostat and a tactile logic device with the composite (Figure 4B).^[^ ^47^ ^]^ In addition, Cho et al. demonstrated an LM–AgNW composite thin film with tunable GF by varying laser‐processing parameters. The group elucidated that as the laser intensity increased when sintering, LM particles ruptured more vigorously and covered a larger portion of the AgNW network. Consequently, GF decreased as the laser intensity increased (Figure 4C).^[^ ^48^ ^]^

## 3 LM‐Patterning Methods

### 3.1 Serial‐Patterning Techniques (Direct Writing)

Serial patterning, or direct writing (DW), can be categorized into three ways: 1) additive, 2) subtractive, and 3) coalescence of LM particles. In this review, “writing” is defined as a pattern that is written serially, not parallelly. Currently, low throughput is the disadvantage of this method,^[^ ^17c^ ^]^ but its precise dynamic controllability for customized design of circuits makes it promising candidate for patterning LM‐based soft electronics.

#### 3.1.1 Additive DW

**Figure 5:**
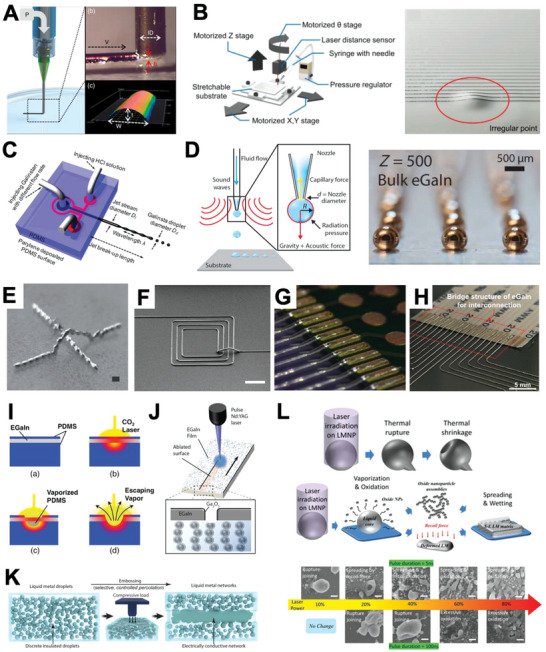
LM serial patterning methods. A) Main parameters of consideration for shear‐driven DW. Reproduced with permission.^[^ ^50^ ^]^ Copyright 2019, Wiley‐VCH. B) Illustration for four degrees‐of‐freedom DW (left), optical image of printed LM on surface with bump (right). Reproduced with permission.^[^ ^52^ ^]^ Copyright 2018, Wiley‐VCH. C) Schematic diagram of inkjet printing system capable of Galinstan droplet generation. Reproduced with permission.^[^ ^58^ ^]^ Copyright 2016, The Royal Society of Chemistry. D) Schematic diagram of acoustophoretic printing (left), optical image of EGaIn droplet generated via acoustophoretic printing (right). Reproduced with permission.^[^ ^61a^ ^]^ Copyright 2018, AAAS. E) Arch structure made of LM droplets. Reproduced with permission.^[^ ^62g^ ^]^ Copyright 2013,Wiley‐VCH. F) Reconfigured LM pattern and the resulting 3D arch‐shaped LM pathway. Reproduced with permission.^[^ ^62h^ ^]^ Copyright 2019, AAAS. G) Magnified view of 3D EGaIn connection. Adapted with permission.^[^ ^62i^ ^]^ Copyright 2019, American Chemical Society. H) 3D bridge‐structure EGaIn connection. Reproduced with permission.^[^ ^51f^ ^]^ Copyright 2020, Wiley‐VCH. I) Drawing of laser‐cutting mechanism for sandwitched‐EGaIn layer within PDMS. Reproduced with permission.^[^ ^63b^ ^]^ Copyright 2014, Wiley‐VCH. J) Schematic drawing of laser ablation of EGaIn on zPDMS composite. Adapted with permission.^[^ ^63c^ ^]^ Copyright 2017, American Chemical Society. K) Mechanical coalescing via compression. Reproduced with permission.^[^ ^39^ ^]^ Copyright 2021, Springer Nature. L) Illustration of thermal rupture‐dominated DC and vaporization‐dominated DC (top), behavior of LM particle under variation of laser power for two different pulse duration condition (bottom). Reproduced with permission.^[^ ^33e^ ^]^ Copyright 2019, Wiley‐VCH.

Additive DW originates from the engineering desire to draw complex electrically conducting patterns using a mere pen similar to conventional art sketching.^[^ ^48^ ^]^ One intuitive DW method is drawing with a roller‐ball pen, which was presented by Zheng et al. The group directly wrote an LM circuit with the pen onto a flexible polymer, forming an electronic device. The width and thickness of the pattern were as low as 200 and 80 µm, respectively. They were able to draw capacitors with various designs.^[^ ^49^ ^]^ For higher LM pattern quality and computer‐aided automation, nozzle‐based additive DW methods have been developed.^[^ ^50^ ^]^ A representative fabrication schematic for nozzle‐based additive DW is demonstrated in **Figure** **5**A.^[^ ^25^, ^50^, ^51^ ^]^ Ink is exerted from a nozzle, which adheres to a substrate while a moving stage or the nozzle induces drawing of a circuit. Such a method can create continuous LM lines with desired diameters by precisely controlling LM adherence to the substrate, nozzle diameter, pressure, movement of nozzle or stage, and standoff distance (SOD; the distance between the nozzle tip and the substrate). Moreover, by maintaining SOD while patterning, this technique can be utilized for printing on bumpy surfaces. To maintain SOD when patterning on an uneven 3D surface, Yoon et al. demonstrated a four‐degrees‐of‐freedom DW system by precise laser‐assisted SOD feedback control. The system was capable of drawing LM lines on wavy elastomer surfaces as shown in Figure 5B.^[^ ^52^ ^]^ When conducting nozzle‐based additive DW of bulk LM, surface interaction is one of most critical factors to consider. For instance, Watson et al. utilized the phenomenon that surface interaction is enhanced when voltage is applied between the substrate and the nozzle to pattern bulk LM on dielectric surface. They investigated the relationship of surface tension and the applied voltage. They were able to create vertically aligned LM patterns on various substrates, obtain different trace widths with the same trace thickness, pattern on omniphobic surfaces, and selectively dip‐coat a substrate utilizing RF circuit.^[^ ^53^ ^]^

Liquid metal composites that have LM droplets within a polymer matrix are also frequently used as ink for nozzle‐based additive DW. For example, Han et al. fabricated thermoelectric generators by printing liquid metal elastomer composites. The group printed liquid metal elastomer composite for thermal interface layer and interconnects to fabricate stretchable thermoelectric wearables.^[^ ^55^ ^]^ Haake et al. demonstrated manipulation of microstructures of liquid metal elastomer composite by modifying the parameters of nozzle‐based additive DW. The group successfully controlled the aspect ratio of the written ink by changing nozzle velocity, resulting in connected droplets for fast velocity and disconnected for slow velocity in a single printing process.^[^ ^56^ ^]^ Neumann et al. presented a self‐encapsulating liquid metal elastomer composite, which was fabricated by nozzle‐based additive DW. The group utilized the phenomenon that heavy LM particles settle by gravity creating a self‐encapsulating structure. The settled part was mechanically sintered to achieve electrical conductivity and utilized as an electrode.^[^ ^36e^ ^]^ Tavakoli et al. created liquid metal elastomer composite that is resilient, repairable, and recyclable. The group mixed reversible polymer, LM, and filler metal microparticles for ink formation. They were able to fabricate various electric devices by patterning the ink and mounting chips.^[^ ^57^ ^]^

Drop‐on‐demand inkjet printing (DoD‐IJP) is another additive DW method for LM patterning. Conventional DoD‐IJP techniques^[^ ^57^ ^]^ are unsuitable for patterning LM owing to its solid oxide layer, which causes clogging inside the inkjet nozzle.^[^ ^58^ ^]^ Therefore, LM droplets for DOD‐IJP should be created using alternative methods such as using a customized nozzle,^[^ ^59^ ^]^ acoustic pulse assistance,^[^ ^60^ ^]^ or modifying LM ink.^[^ ^35^ ^]^ For instance, Li et al. fabricated DoD‐IJP equipment utilizing multiple microfluidic channels. They injected one channel with Galinstan and the other channel with 37 wt% HCl solution. The acidic solution removed oxide on Galinstan, which facilitated printing^[^ ^58^ ^]^ (Figure 5C). Foresti et al. utilized acoustophoretic force to create LM droplets for DOD‐IJP. Localized acoustophoretic force generated by sound with a frequency of ≈20 kHz caused ejection of LM droplets of uniform radius from the nozzle orifice^[^ ^61a^ ^]^ (Figure 5D). Ink modification can be another solution for creating LM droplets for DOD‐IJP. For example, Boley et al. fabricated thiol (3‐Mercapto‐N‐nonylpropionamid)‐grafted LM nanoparticles by sonication. The thiol decreased the effective surface tension of the particle, which facilitated successful DOD‐IJP.^[^ ^35^ ^]^

Additive DW can also be utilized to create 3D LM structures.^[^ ^61^ ^]^ For instance, Ladd et al. fabricated freestanding LM structures at room temperature. The group determined that the pressure of EGaIn inside the surrounding oxide should be maintained ≈4 kPa to form pillar‐shaped wires without collapsing during elongation. With careful control of the pressure exerted on the wire, freestanding LM pillars as long as 1 cm were realized. Furthermore, the group successfully created a freestanding LM arch, tower, cubic in the same research^[^ ^61^ ^]^ (Figure 5E). Park et al. fabricated a 3D LM wire structure and utilized it as an interconnection between circuit electrodes. The group demonstrated that 2D LM patterns could be lifted in the vertical direction and then transported to another substrate through precise nozzle velocity and pressure control. The group realized forming a 3D arch‐shaped conductive pathway^[^ ^62h^ ^]^ (Figure 5F). Similarly, Kim et al. fabricated a 3D LM wire connection on various metal electrodes using DW. LM wires were extended, lifted, and stretched between metal electrodes, maintaining a bridge‐like 3D geometry. The group reported that the LM bridge showed high mechanical and thermal stability (Figure 5G).^[^ ^61^ ^]^ Such a bridge‐like LM interconnection was also utilized in the work of Oh et al. The group patterned a thermal and tactile sensor feedback circuit via DW and connected a flexible flat cable with the circuit by 3D LM wires that were generated by DW^[^ ^50^ ^]^ (Figure 5H).

#### 3.1.2 Subtractive DW

The subtractive DW method forms an LM pattern by removing the unwanted portion and leaving only the desired portion.^[^ ^62^ ^]^ In most cases, subtractive DW is performed by exposing a target to a high‐energy laser, which induces cutting or direct ablation. For example, Lu et al. proposed a CO_2_ laser‐based cutting method. The group cut a PDMS‐embedded LM layer for obtaining the desired pattern. When cutting with laser, they found that rapid Ga_2_O_3_ layer formation right after the laser exposure prevented leakage of adjacent LM from the cutting surface^[^ ^62^ ^]^ (Figure 5I). By the way, Lu et al. performed direct laser ablation of an LM film using an ultraviolet laser micromachining (UVLM) system. They first deposited LM on a modified PDMS substrate with a roller. Then, laser ablation was conducted on the undesired portion, leaving only the desired portion after the process. The group was able to fabricate a pulse oximeter with the laser‐ablated LM circuit^[^ ^62^ ^]^ (Figure 5J). Similarly, Pan et al. fabricated a visually imperceptible LM circuit with laser ablation. In this research, the laser ablation was performed on PDMS with a Cr/Cu/LM layer deposited. The fabricated grid circuit was as thin as 19 µm, realizing a transparent circuit.^[^ ^62^ ^]^

#### 3.1.3 Direct Coalescence

As mentioned in Section 2.3, LM particles without sintering do not possess electrical conductivity. Therefore, researchers have patterned the particles into an electrical circuit by selectively sintering them. Herein, such a patterning method is called “direct coalescing (DC).” Mechanical DC is a simple method that selectively sinters LM particles with mechanical stress.^[^ ^36^, ^39^, ^64^ ^]^ For instance, Boley et al. demonstrated selective mechanical DC on an LM film using a Si needle. They also analyzed the force required to sinter a single LM particle with different sizes, where a positive relationship between the force and particle size was observed.^[^ ^36b^ ^]^ Similarly, Li et al. performed mechanical DC using a diamond tip. The group successfully generated an LM pattern on rough paper by depositing LM and mechanically sintering with the tip.^[^ ^64c^ ^]^ In addition, mechanical DC can be applied to LM composites. For example, Tutika et al. fabricated an LM composite made of styrene–isoprene–styrene (SIS), LM particles, and polybutadiene. LM patterns could be created by sintering LM particles inside the composite by mechanical embossing (Figure 5K).^[^ ^39^ ^]^ Similarly, Markvicka et al. also demonstrated that mechanical DC can be applied to a composite composed of LM microparticles and PDMS.^[^ ^64d^ ^]^ Both composites composed of SIS and PDMS exhibited unique damage tolerance, which was attributed to spontaneous sintering while being damaged.

DC can also be performed by using a laser.^[^ ^33^, ^65^ ^]^ Contrary to laser cutting or ablation, laser can be used for sintering if appropriate power and wavelength are selected. For example, Liu et al. used a 1065‐nm fiber laser to coalesce sprayed LM nanoparticles to form conductive lines. The laser‐sintered LM film had a distinctive feature that only the upper part of the film was sintered. The group was able to fabricate a stacked circuit with the laser‐sintered film utilizing that feature.^[^ ^33b^ ^]^ Similarly, Deng et al. fabricated an LM circuit using laser DC. The group revealed that there were two different mechanisms in laser DC—thermal rupture‐ and vaporization‐dominated mechanism—depending on the laser power. They explained these two mechanisms as follows. 1) Thermal rupture‐dominated DC occurs when a weak laser induces thermal expansion and, eventually, rupture of LM particles. 2) By contrast, vaporization‐dominated DC occurs when a strong laser induces vaporization of the core in LM particles, which destroys the shell oxide and spreads the nonvaporized core to the substrate. When the vapor cools down in air and reattaches to the substrate, it becomes a metal oxide nanoparticle, which serves as a seed layer for wetting nonvaporized LM, which promotes solid–liquid dual phase pattern with repeated laser exposure^[^ ^33e^ ^]^ (Figure 5L).

### 3.2 Parallel‐Patterning Techniques (Parallel Printing)

Parallel printing methods are suitable for fabricating LM patterns over a broad area in a parallel manner. In this subsection, representative examples of LM printing will be introduced, namely, stencil printing and transfer printing. It not only realizes high reproducibility of complex patterns due to its high throughput property and relatively simple process, but also high‐resolution conductive pathways. However, they suffer from cost issue because different patterns require different stencils or stamps.

#### 3.2.1 Stencil Printing

**Figure 6:**
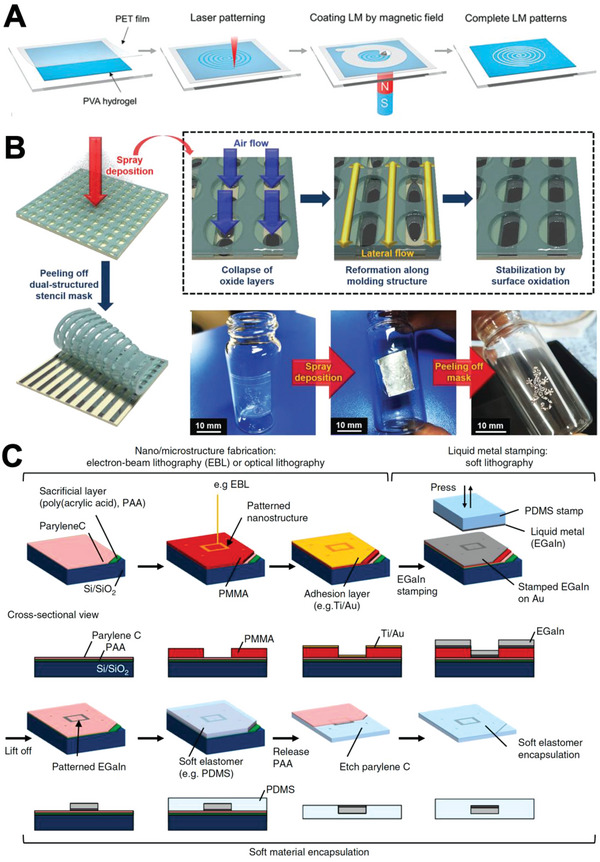
LM parallel patterning methods–stencil printing and lift‐off. A) Illustration of magnetic field‐assisted stencil printing process. Reproduced with permission.^[^ ^66^ ^]^ Copyright 2019, Wiley‐VCH. B) Schematic illustration of process of LM patterning with dual‐structured stencil (top), LM patterned on curved surface (bottom right). Reproduced with permission.^[^ ^70^ ^]^ Copyright 2020, Wiley‐VCH. C) Illustration of lift‐off process assisted by electron‐beam/soft lithography. Reproduced with permission.^[^ ^72a^ ^]^ Copyright 2020, Springer Nature.

LM patterns can be created by blade‐coating LM over a stencil.^[^ ^66^ ^]^ However, achieving stable patterns is generally difficult because of the high surface tension of pure LM. Various LM ink modifications have been proposed to solve the problem. For instance, Xu et al. suggested a magnet‐assisted coating of LM ink mixed with nickel microparticles. The group used a laser‐patterned PET mask to stencil‐print LM onto a polyvinyl alcohol hydrogel substrate with the modified ink^[^ ^67^ ^]^ (**Figure** **6**A). Similarly, Liu et al. created biphasic LM ink by the thermal sintering (900 °C) of pure LM, followed by cooling. The ink comprised solid *β*‐Ga_2_O_3_ crystal particles and LM. The group successfully stencil‐printed the ink with a mask on acrylic tape and silicone elastomer.^[^ ^43b^ ^]^ Cao et al. fabricated ferromagnetic liquid metal composite ink that can transform shape and reconfigure polarity. The group mixed ferromagnetic neodymium–iron–boron microparticles with LM to achieve it. They stencil‐printed the ink and successfully controlled its magnetic polarity by a magnet.^[^ ^68^ ^]^

As an alternative way for blade‐coating LM, spraying LM aerosol ink over a stencil has been attempted.^[^ ^36^, ^69^ ^]^ LM aerosols are typically manufactured by effusing LM ink with the assistance of carrier gas flow.^[^ ^70^ ^]^ For example, Guo et al. reported that LM with a 30% NaOH solution could be sprayed to generate conductive patterns on pig skin with stencil.^[^ ^69c^ ^]^ Similarly, Zhang et al. sprayed pure LM over a mask using an air nozzle and patterned LM lines on various substrates. The conductivity was obtained as soon as it was deposited on the substrate due to the high air pressure and close distance while spraying. The group demonstrated that patterning LM on a 3D‐structured substrate was also possible.^[^ ^69b^ ^]^ To enhance LM pattern quality, Park et al. demonstrated spray‐printing of LM with a dual‐structured polyurethane acrylate stencil. The stencil comprised an upper portion that served as a support and a lower portion that served as a mold for LM ink. The stencil realized fine LM patterning on nonplanar surfaces without any leakage^[^ ^71^ ^]^ (Figure 6B).

Lift‐off methods are frequently performed in a similar manner with stencil printing where photoresist substitute stencil.^[^ ^22^, ^71^ ^]^ For example, Park et al. spin‐coated a negative photoresist and patterned it using photolithography. Then, LM was spread over the entire substrate. Finally, lifting off the photoresist by development realized LM patterning on the substrate.^[^ ^71^ ^]^ Similarly, Kim et al. used electron‐beam lithography to engrave a pattern on a photoresist for achieving high pattern resolution. Then, a Ti/Au adhesion layer was constructed on the photoresist, and LM was deposited. Finally, the photoresist was lifted off to form the LM pattern, and the pattern was transferred to PDMS^[^ ^71^ ^]^ (Figure 6C).

#### 3.2.2 Transfer Printing

Transfer printing (TFP) is defined as a technique that is capable of designing LM patterns by transferring the LM pattern from a primary substrate to a target secondary substrate.^[^ ^73^ ^]^ TFP is realized in a parallel manner. Relief stamping, subtractive stamping, dual‐transfer printing, and thermal transfer printing are the representative examples of TFP.

**Figure 7:**
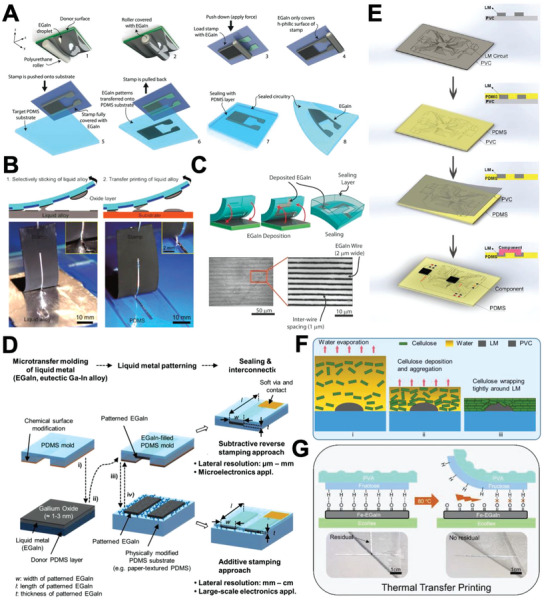
LM parallel patterning methods – TFP (transfer printing). A) Illustration of process for stamp‐printing of EGaIn followed by encapsulation. Reproduced with permission.^[^ ^75^ ^]^ Copyright 2019, Wiley‐VCH. B) Stamp printing enabled by selective LM wetting onto cPDMS with modified surface roughness. Adapted with permission.^[^ ^19a^ ^]^ Copyright 2019, American Chemical Society. C) EGaIn filling the engraved part of the stamp and being encapsulated (top), the resulting LM lines (bottom). Reproduced with permission.^[^ ^77^ ^]^ Copyright 2014, Wiley‐VCH. D) Illustration of additive/subtractive stamping. Reproduced with permission.^[^ ^76b^ ^]^ Copyright 2018, Wiley‐VCH. E) Dual‐trans printing process. Reproduced with permission.^[^ ^19b^ ^]^ Copyright 2015, Wiley‐VCH. F) Evaporation‐induced transfer printing. Reproduced with permission.^[^ ^19c^ ^]^ Copyright 2021, Elsevier. G) Thermal transfer printing. Reproduced with permission.^[^ ^18^ ^e]^ Copyright 2019, Wiley‐VCH.

Stamping is a simple TFP, which is performed by transferring ink from a stamp to a substrate.^[^ ^74^ ^]^ By modifying the stamp to allow selective adherence of the LM ink to the patterned surface, high‐quality LM patterns can be achieved. For instance, Yalcintas et al. performed TFP using a surface‐modified PDMS stamp. LM ink selectively wetted on a relief structure that was modified with an LM‐favorable NTPE (N‐[3‐(Trimethoxysilyl)propyl]) self‐assembled monolayer, while the other part was covered with LM‐unfavorable TFOCS (Trichloro(1H,1H,2H,2H‐perfluooctyl)). The group successfully fabricated a microcapacitive sensor and microresistor with the stamp (**Figure** **7**A).^[^ ^75^ ^]^ Altering surface morphology of the stamp is another way to achieve selective adherence. For example, Zhang et al. selectively UV‐treated a carbon‐dosed silicone (cPDMS) stamp, creating a rough and lyophobic surface. The selective wetting of Galinstan on the elastomer stamp realized printing on a complex 3D surface. The group fabricated an LM pattern with a line width of 20 µm and line spacing of 40 µm with a short working time of 82 min (Figure 7B).^[^ ^19a^ ^]^

When patterning with a stamp, LM spread on the relief part is usually utilized for patterning. Nevertheless, some studies have also utilized LM smeared into the engraved part.^[^ ^76^ ^]^ For instance, Gozen et al. achieved high‐resolution LM patterning with line width and spacing as small as 2 and 1 µm, respectively, using a PDMS mold. The process was performed in three steps. First, LM was filled into a microchannel by stamping the mold onto the LM reservoir. Second, the LM ink adhered to relief structures was removed by stamping onto the sacrificial substrate. Third, the stamp with its mold filled by LM was encapsulated (Figure 7C).^[^ ^77^ ^]^ Kim et al. suggested a “multiscale” subtractive and additive stamping method that utilized PDMS mold‐filled LM as well as residue LM on the relief part for additive stamping. Such an approach realized patterning of two different patterns using a single stamp (Figure 7D).^[^ ^76b^ ^]^

The dual‐transfer method is utilized when an LM circuit needs to be embedded in an elastomer. For instance, Wang et al. performed dual‐transfer of the LM pattern from PVC to PDMS. The secondary substrate—PDMS—covered the LM pattern on PVC and cured. Then, the entire system was frozen to solidify the LM pattern, which facilitated peeling off the PVC layer while leaving the pattern on PDMS. Thus, the LM pattern was successfully transferred to PDMS with no significant change (Figure 7E).^[^ ^19b^ ^]^ Similarly, Mao et al. reported evaporation‐induced transfer printing that used the mechanical wrapping force of nanocellulose. LM was previously patterned on a sacrificial PVC substrate, and nanocellulose solution was spread over the pattern. The pattern was successfully transferred to a nanocellulose membrane by the wrapping force induced by evaporation (Figure 7F).^[^ ^19c^ ^]^

Thermal transfer printing utilizes ink adherence modification by heating.^[^ ^78^ ^]^ For example, Guo et al. reported thermal transfer printing of Fe microparticles mixed into GaIn (Fe–EGaIn) integrated with DW of fructose using a ball‐point pen. The group demonstrated that Fe–GaIn ink selectively adhered to prepatterned fructose via hydrogen bonding. As shown in Figure 7G, successful transfer of LM pattern from polyvinyl alcohol (PVA)/fructose to Ecoflex was achieved by heating them at 80 °C. The heat reduced the adherence between LM and fructose by breaking the hydrogen bonds, leaving almost no residual LM on the PVA/fructose after the retrieval.^[^ ^19d^ ^]^

### 3.3 Molding and Microfluidic Injection

**Figure 8:**
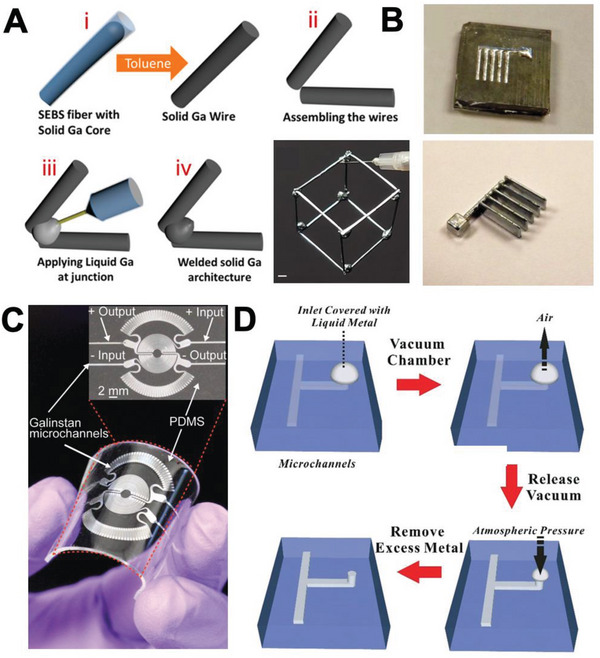
LM molding and microfluidic injection. A) Ga wire fabrication and wire welding (top), 3D structure design via welding Ga wires (bottom right). Adapted with permission.^[^ ^81a^ ^]^ Copyright 2021, American Chemical Society. B) 3D LM structure fabrication via EGaIn molding. Reproduced with permission.^[^ ^81c^ ^]^ Copyright 2013, The Royal Society of Chemistry. C) Pressure sensor fabricated via microfluidic injection of LM. Reproduced with permission.^[^ ^82f^ ^]^ Copyright 2017, Wiley‐VCH. D) Vacuum filling LM into microchannel. Reproduced with permission.^[^ ^83^ ^]^ Copyright 2017, The Royal Society of Chemistry.

Fabricating an LM circuit is possible by filling LM into prepatterned channels.^[^ ^79^ ^]^ Molding and microfluidic injection are the representative examples. They are widely used in the fields of soft electronics because of their simple process for generating elastomer‐embedded conductive tracks. However, they also suffer from relatively low pattern resolution due to high surface tension of LM. In the case of LM molding, LM fills the engraved pattern of mold and then is either encapsulated by elastomer or freeze‐casted to be separated from the mold.^[^ ^80^ ^]^ Moreover, 3D LM structures can also be fabricated by freeze‐casting LM from molds.^[^ ^81^ ^]^ For example, Bhuyan et al. fabricated Ga wires by encapsulating Ga inside a styrene–ethylene–butylene–styrene (SEBS) hollow fiber, followed by solidification of LM and etching of SEBS with toluene. This procedure created a freestanding Ga wire. The group constructed various 3D structures using Ga wires, where Ga droplets were utilized for connecting junctions (**Figure** **8**A).^[^ ^81a^ ^]^ Similarly, Fassler et al. also fabricated 3D LM structures by combining freeze‐casting and vacuum casting. First, the group fabricated a surface‐patterned elastomer deposited with an LM film over it and placed the elastomer in a vacuum for the film to smear into the pattern. Then, they froze the LM‐filled elastomer and safely removed the elastomer, leaving only the 3D LM structure. Finally, the structure was assembled into complex 3D circuits with other structures (Figure 8B).^[^ ^81c^ ^]^

Microfluidic injection methods enable fabrication of LM‐filled channels with complex structures. A typical process of microfluidic injection into an inlet–outlet‐channel is demonstrated in Figure 8C. Applying appropriate pressure to the LM‐filled syringe connected to a channel results in LM to be injected into the channel until it fills the entire system.^[^ ^82^ ^]^ However, negative pressure is required to fill LM into the pore structure or one‐opening channels. For instance, Lin et al. reported a vacuum‐filling method to fill a dead‐end PDMS microchannel by removing air inside the channel and then applying pressure to it to inject LM (Figure 8D).^[^ ^83^ ^]^

When filling LM inside a channel or a mold, difficulty usually arises because of the stickiness of Ga oxide and non‐Newtonian behavior of LM.^[^ ^84^ ^]^ To overcome this adversity, Li et al. proposed two methods to reduce wettability between a surface and LM. The key was modifying the surface. The first method involved fabricating a PDMS microchannel with a paper‐textured inner surface and applying solid nanoparticles such as TiO_2_, carbon nanotube, graphene, or graphite on the textured surface to further increase the roughness. The other method involved treating the channel with various acidic solutions such as H_2_SO_4_, HF, or HNO_3_ to create rough inner surfaces, where 89 wt% H_2_SO_4_ treatment showed the best nonwetting results.^[^ ^19f^ ^]^ Similarly, Khan et al. reported that prefilling a microchannel with deionized water realized smooth LM movement. The group suggested that water not only chemically modified the oxide layer but also formed a slip layer between the inner surface and LM, which prevented the oxide layers from adhering to the channel.^[^ ^85^ ^]^

### 3.4 Intermetallic Bond‐Assisted Patterning

**Figure 9:**
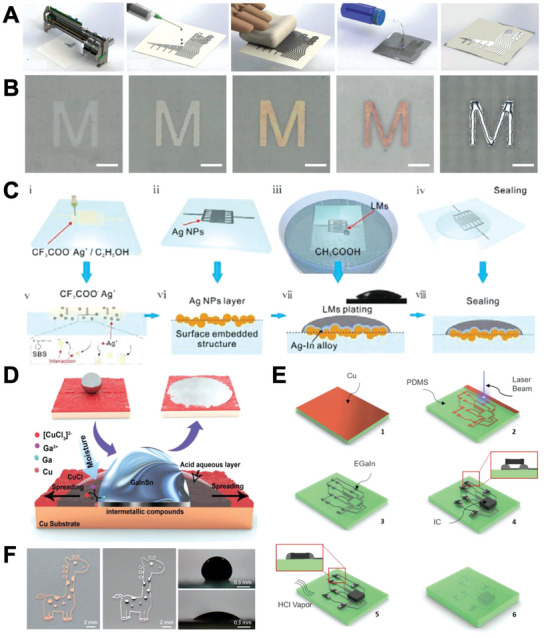
Intermetallic bonding assisted patterning of LM. A) Patterning process for generating EGaIn/AgNP line. Reproduced with permission.^[^ ^88^ ^]^ Copyright 2018, Wiley‐VCH. B) LM/Cu/AgNP pattern generation process assisted by prepatterned PDA (polydopamine). Reproduced with permission.^[^ ^16c^ ^]^ Copyright 2021, Springer Nature. C) Schematic drawings of surface‐embedded AgNP layer structure generation process followed by LM adhesion onto it. Reproduced with permission.^[^ ^89^ ^]^ Copyright 2022, Wiley‐VCH. D) Schematic illustration of Galinstan flattening on Cu sheets. Reproduced with permission.^[^ ^90^ ^]^ Copyright 2021, Wiley‐VCH. E) Manufacturing process of LM/Cu pattern and circuit component integration. Reproduced with permission.^[^ ^93^ ^]^ Copyright 2018, Wiley‐VCH. F) LM adhered onto prepatterned Cu (left), LM wettability difference between with/without Cu film (right). Reproduced with permission.^[^ ^94^ ^]^ Copyright 2022, AAAS.

Because LM favorably alloys with other metals, methods utilizing other patterned metals directly by creating metal alloys have been developed. Such method not only tend to increase electrical conductivity compared to pure LM, but also selective wetting of LM onto prepatterned substrate which can realize high‐resolution circuits.^[^ ^85^ ^]^ The processes for intermetallic bond‐assisted patterning is as follows: 1) an interfacial metal layer is prepatterned, 2) LM is deposited over the layer, 3) acidic/basic solution is applied to LM in order to remove surface oxide accelerating intermetallic bonding, and 4) rinsing of LM residue. For instance, Dejace et al. sputtered Au onto patterned PDMS and removed Au on the relief structure by ion‐beam etching. Thermally evaporated Ga selectively adhered to the Au remaining on the gravure of PDMS. Accordingly, an transparent electronic circuit was successfully fabricated.^[^ ^86^ ^]^ Similarly, Tavakoli et al. inkjet‐printed silver nanoparticle ink onto tattoo paper and then applied LM over the pattern to fill the voids between silver nanoparticles to achieve conductivity. Selective LM adhesion was possible owing to LM–Ag alloy formation. The residue was cleaned by treating with an acidic solution. The patterned LM–Ag alloy was stretchable up to 110% strain with stable electrical connection (**Figure** **9**A).^[^ ^87^ ^]^ Zhu et al. proposed an electroless plating method to form a prepatterned Cu layer for intermetallic bond‐assisted patterning. The group prepatterned polydopamine (PDA) by stencil printing and removed the stencil using ethyl alcohol. PDA acted as a reducing agent to form silver nanoparticle (AgNP) inside the AgNO_3_ solution, where AgNP assisted the Cu film in growing into the same pattern of PDA. After the process, Galinstan selectively wetted Cu lines with the assistance of HCl, which reduced Galinstan's oxide (Figure 9B).^[^ ^15^ ^]^ Liu et al. showed that Galinstan selectively wetted AgNP lines and formed AgIn_2_ alloy by submerging the entire system into 5 wt% acetic acid solution. The AgNP lines were prepatterned on poly(styrene‐*b*‐butadiene‐*b*‐styrene) (SBS) by reducing Ag^+^ with hydrazine hydrate (NH_2_NH_2_·H_2_O) vapor. Subsequent LM deposition and selective wetting completed the patterning procedure (Figure 9C).^[^ ^88^ ^]^ Lin et al. reported that when CuCl_2_ solution was added to a rough Cu layer, Galinstan strongly wetted the Cu layer because Galinstan underwent several chemical reactions. A circuit was fabricated by spraying CuCl_2_‐treated Cu powder over a patterned adhesive tape layer and then spreading Galinstan over them. The group also found out that Galinstan successfully wetted CuCl_2_‐treated rough Ni and Fe layers (Figure 9D).^[^ ^89^ ^]^ Ma et al. demonstrated electrochemical manipulation of LM to wet porous copper. The manipulation was conducted in the NaOH solution with a certain DC voltage. The group cut PVC into desired design to pattern LM utilizing this wetting behavior, and conducted electrochemical coating and subsequent wiping.^[^ ^90^ ^]^

Because a firm connection between an elastomer and prepatterned metal is important in intermetallic bond‐assisted patterning, researchers have developed a technique to achieve such connections. The technique is to lay a Cr layer before depositing the interfacial metal film on an elastomer.^[^ ^16^, ^92^ ^]^ For instance, Ozutemiz et al. deposited a Cr/Cu film on an elastomer and patterned the film by UV laser ablation. The group achieved a patterned LM film by submerging the LM and the patterned metal film together in 3 wt% NaOH solution, inducing intermetallic bonding. They successfully created a complete LM circuit with surface‐mounted electrical components (Figure 9E).^[^ ^93^ ^]^ Similarly, Wang et al. subtractive‐patterned Cr/Cu lines on an SEBS substrate by laser ablation and then spread LM over the prepatterned system. The process was realized inside HCl solution, which allowed LM to lose its oxide layer and selectively wet the metal pattern (Figure 9F).^[^ ^94^ ^]^

Furthermore, a surface‐structured metal underlayer can create a smooth LM surface, which generally possesses stable electromechanical properties. For example, Hirsch et al. designed an Au‐filmed PDMS with mesh‐like microstructures to create a uniform‐height Ga pattern. The group performed thermal evaporation to deposit Ga on the PDMS and obtained the uniform‐height pattern. The smooth pattern acquired from mesh‐like microstructured PDMS demonstrated stable electromechanical response under strain, whereas a pattern acquired from flat PDMS did not.^[^ ^95^ ^]^ Similarly, Kim et al. reported that a smooth and large‐area LM film could be fabricated by designing Cu‐deposited micropillar‐array structures. Governed by the imbibition phenomenon, LM autonomously spread onto and wetted the microstructure, while oxide was removed by HCl vapor. The group successfully fabricated an LM/Cu/PDMS composite with a smooth and thin LM surface.^[^ ^96^ ^]^

## 4 Perspective and Conclusion on LM‐Based Future Soft Electronics with Unique Properties Incorporated with Patterning

### 4.1 Outstanding Features of LM‐Based Soft Electronics Incorporated with Patterning and Limitations

#### 4.1.1 Conformability

**Figure 10:**
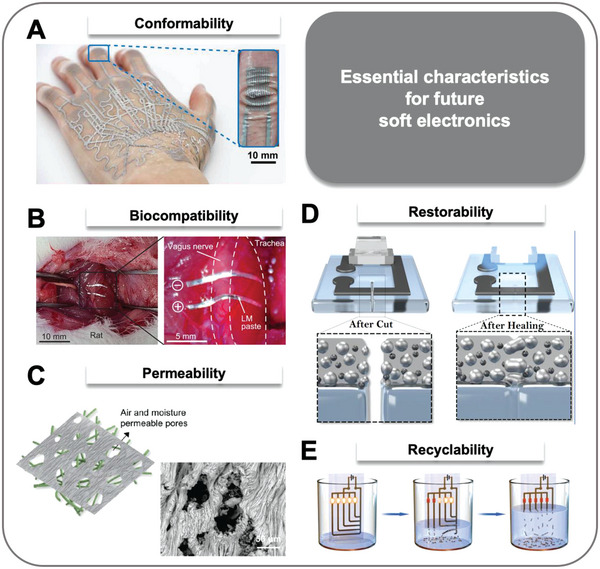
Future perspectives on LM‐based future wearable electronics with unique properties. Image for “conformability”. A) LM‐based wearable electronics on human hand. Reproduced with permission.^[^ ^101^ ^]^ Copyright 2021, AAAS. Image for “biocompatibility”: B) LM electrodes adhered onto rat nerve. Adapted with permission.^[^ ^108^ ^]^ Copyright 2022, American Chemical Society. Image for “permeability”. C) Air‐permeable LM‐based composite. Reproduced with permission.^[^ ^112^ ^]^ Copyright 2021, Wiley‐VCH. Image for “restorability”. D) Restorable conductivity enabled by self‐healing process. Reproduced with permission.^[^ ^115c^ ^]^ Copyright 2021, Springer Nature. Image for “recyclability”. E) Degradation of LM circuit encapsulated with PVA by dipping into water. Reproduced with permission.^[^ ^120^ ^]^ Copyright 2019, Wiley‐VCH.

Conformability is a primary requirement for a comfortable and unobtrusive soft device.^[^ ^97^ ^]^ The device should be highly deformable, thin, and adhesive to attain conformability owing to the roughness of soft surface.^[^ ^98^ ^]^ The fluidic nature of LM enables it to spread thinly on a substrate while possessing freely deformable form.^[^ ^99^ ^]^ Thus, LM is an appropriate material for soft devices with high conformability when incorporated with a thin and adhesive substrate.^[^ ^100^ ^]^ For instance, Tang et al. attached LM‐based electronic tattoos to human skin using an extremely thin SBS substrate. Such tattoos exhibited conformal contact even on bumpy surfaces of the human skin such as fingerprints. The group successfully fabricated a multilayered electronic transfer tattoo through repeated deposition of the substrate and LM circuit (**Figure** **10**A).^[^ ^101^ ^]^ Pei et al. synthesized a thioctic‐copolymer‐grafted LM elastomer composite, which was capable of forming wet adhesion on various substrates, including the human skin. It adhered stably to finger joints and biceps and facilitated on‐skin EMG detection successfully.^[^ ^102^ ^]^

#### 4.1.2 Biocompatibility

Biocompatibility is essential for wearable electronic devices to be applied directly to a living creature's body such as a skin or an organ.^[^ ^102^ ^]^ Although it was reported that cytotoxic Ga and In ions tend to be generated in large amount when sonication is done under aqueous environment condition,^[^ ^103^ ^]^ most of the soft electronic device operations or patterning process do not frequently encounter such drastic stimulation, which promises negligible cytotoxicity. Such advantage of LM can be fully utilized with high conductivity, stretchability, and conformability for nontoxic wearable/implantable devices.^[^ ^104^ ^]^ LM‐based wearable devices have been fabricated with various biocompatible materials such as polycaprolactone, polyacrylamide‐silk fibroin hydrogel, gluten, and SBS.^[^ ^29^, ^93^, ^105^ ^]^ For instance, Wang et al. demonstrated the adhering of oxidized GaIn with metal oxide particles (O‐GaIn) to bare mouse skin. The O‐GaIn tattoo was directly applied to a tumor in the mouse's back. The wireless power transfer to the tattoo realized electromagnetic heating for in vivo tumor therapy.^[^ ^106^ ^]^ Similarly, Murakami et al. demonstrated that LM–Ni composites that were previously patterned on a PVA film could be successfully transferred onto the vagus nerve of a rat. The transfer was performed by dissolving the PVA film with water while attaching the LM composite circuit to the target surface. The group reported that the nerve was successfully stimulated by inducing an electrical current in the LM electrodes (Figure 10B).^[^ ^107^ ^]^ Wang et al. reported the successful attachment of an LM‐based sensor to a rabbit's heart using polydopamine–polyacrylamide hydrogel adhesive. The sensor stably obtained a signal from the movement of the heart.^[^ ^93^ ^]^

#### 4.1.3 Permeability

Permeable electronics are desirable for nonirritating and conformal wearable devices.^[^ ^109^ ^]^ An unbreathable device induces human skin to be wetted by sweat or other body‐originated fluids, which induces inflammatory skin responses such as erythema or rash while decreasing conformability.^[^ ^106b^ ^]^ To overcome this limitation, porous substrates such as electrospun fiber substrates have been used to fabricate highly breathable devices.^[^ ^110^ ^]^ Highly conductive and stretchable porous devices can be realized when such substrates are incorporated with LM.^[^ ^111^ ^]^ For example, Ma et al. fabricated an LM–SBS composite with high stretchability, conductivity, and permeability. By performing repeated stretching and releasing up to 1800% strain, highly porous structures were generated while LM was simultaneously coated along the SBS fiber. The group reported that no skin irritation occurred while the composite was worn on human skin for one week.^[^ ^30^ ^]^ Similarly, Zhuang et al. performed modification on an SBS fiber substrate by adding an Ag layer for enhanced wettability of LM onto the fiber‐based substrate. The fluidic nature of LM could wet the breathable substrate with high affinity via continuous stretching and releasing. The group fabricated a permeable electrical circuit and heater patch with the composite (Figure 10C).^[^ ^112^ ^]^ Cheng et al. fabricated a permeable LM–PEG–PDMS composite that strongly adhered to human skin. Sufficient permeability could be obtained when the thickness of the composite was less than 100 µm. The composite did not show any skin reactions while stably adhering onto the forearm for 48 h under extreme activities such as exercise and washing.^[^ ^113^ ^]^

#### 4.1.4 Restorability

Electrical interconnections in soft devices are vulnerable to external damage, such as extreme deformation, friction, or impact. By exploiting restorable electronics that have a self‐healing property, stable operation under various external stimuli can be realized.^[^ ^105^, ^114^ ^]^ LM is a suitable material for restorable electronics owing to its liquid‐originated self‐healing nature.

Unlike other rigid metals, LM has the ability to coalesce without additional treatment when it comes in contact because it is fundamentally a liquid.^[^ ^43c^ ^]^ This property realizes restorable electronics as the breakage of electrical circuit by external stimuli can be self‐healed autonomously when the contact between broken parts is available. Various LM‐based composites possessing restorability have been proposed.^[^ ^19^, ^39^, ^64^, ^67^, ^115^ ^]^ For instance, Lopes et al. printed Ag–LM–SIS ink on an SIS substrate to create an electrical circuit. To demonstrate restorability, the group intentionally cut the composite. Restoration was accomplished by treating the composite with toluene vapor, which induced pol–gel transition of the ink as well as the substrate because toluene could dissolve them simultaneously. After the vapor was removed, the ink and the substrate reattached, showing negligible difference with the pristine composite (Figure 10D).^[^ ^115c^ ^]^ Similarly, Xu et al. patterned LM/Ni microparticle composite ink on a polyvinyl alcohol hydrogel substrate. Upon cutting, the hydrogen bonding between the cut surfaces of polyvinyl alcohol hydrogel led to autonomous self‐healing, and two separated LM paths were connected owing to the liquid property of the LM. The group reported that complete restoration was realized when sufficient healing time of 24 h was provided, where the self‐healed conductive line exhibited almost negligible variation in electromechanical behavior compared to the original one.^[^ ^67^ ^]^

#### 4.1.5 Recyclability

Recyclability prevents wastage of resources when discarding or redesigning electronic devices, thereby realizing economic usage of limited materials.^[^ ^116^ ^]^ Because LM exists as a liquid with high surface tension at room temperature, this property facilitates extracting LM from an LM‐based composite.^[^ ^117^ ^]^ Once the LM is separated from other materials via a certain solution process, it tends to merge together because of high surface tension. After merging, a simple extraction of the merged LM sphere affords recycling. When LM is utilized with other recyclable materials, entirely recyclable electronics become feasible.^[^ ^118^ ^]^

Several studies on recyclable LM‐based circuits have been conducted.^[^ ^39^, ^114^ ^]^ For example, Li et al. proposed an LM paper composed of cellulose nanofibrils, PVA, and LM by casting them into a mold. The paper could be recycled by simply dissolving the constituents into water and casting them onto a new LM paper.^[^ ^119^ ^]^ Similarly, Teng et al. proposed recyclable electronics made of Galinstan encapsulated by PVA. Degradation of the entire circuit was possible by dipping it in water, and reduction of LM was achieved by adding NaOH solution (0.6 m) to prevent sticking of LM residue onto the inner wall of the container. The group recollected 96% of the original LM in bulk form (Figure 10E).^[^ ^120^ ^]^ Tutika et al. recycled an SIS–PBD–LM composite by shear‐mixing it in toluene and recasting. The recycled composite followed a stress–strain curve different from that of the original composite, with less rigidity. Despite stiffness variation, the recycled composites were highly stretchable up to 1000% strain. In addition, the group confirmed that conductive lines can be reformed using recycled composites.^[^ ^39^ ^]^

#### 4.1.6 Limitations

There are several limitations of LM‐based soft electronics that has to be overcome other than patterning. First, due to the high adhesion of LM oxide, precise wetting control in nanoscale is challenging.^[^ ^120^ ^]^ Second, when forming electrical connection between LM and metallic circuit components, LM leakage at the interface or circuit corrosion by intermetallic reaction can bring about short circuit or impedance variation.^[^ ^121^ ^]^ Third, the fluidity of LM makes it impossible to apply general soldering methods for mounting electronic components into a circuit.^[^ ^114^ ^]^ Such limitations should be surmounted by surface treatment, encapsulation, elastomer selection, alloying or forming composite, or adhesive glue as well as the development of patterning methods for LM.^[^ ^16^, ^97^, ^108^, ^116^, ^122^ ^]^

### 4.2 Perspectives on Future LM‐Based Soft Electronics in Various Areas

#### 4.2.1 Soft RF Electronics

RF electronics is a kind of electronics for realizing high frequency and speed communication technology, which can be applied to various fields requiring long‐distance and precise real‐time signal transmission.^[^ ^54^, ^124^ ^]^ Utilizing LM in RF electronics makes the device soft which is expected to dedicate 5G communications, internet of things, and healthcare monitoring technology. One of challenges when fabricating RF device stems from the adhesion between substrate and conductor. RF laminates—a group of high dielectric performance polymers used as substrates for RF circuit—are mainly used for its benefits on realizing high signal quality.^[^ ^124^ ^]^ Unfortunately, such materials are difficult to be integrated with LM circuit due to low surface energy and roughness, which lower patterning quality and eventually leads to unreliable electrical performance.^[^ ^54^ ^]^ Another challenge is achieving high surface smoothness of conductive path. In 5G communication, rough conductor surface can enlengthen the conductive path and reduce the skin depth, which results in low performance of the circuit.^[^ ^124^ ^]^ LM can be an effective solution to these challenges due to its high surface tension induced smooth surface and liquid‐originated various patterning techniques mentioned in this review. For example, Watson et al. presented electrowetting‐assisted LM patterning technique which successfully realized stable LM pattern onto wetting‐unfavored substrates and controlled pattern width by tuning applied voltage while maintaining constant thickness. Furthermore, the group demonstrated that such voltage‐assisted DW method could create 3D LM structures on SiO_2_, parylene C, and methyltrichlorosilane SiO2 substrate, which presented its potential application for 3D RF electronics.^[^ ^54^ ^]^ Yamagishi et al. reported smooth‐injection of Galinstan into silicone microchannels placed between ECOflex and PVA layers that were spaced as small as 7 µm by applying temporary PVA layer. LM thin‐channel injection technique not only enabled microthickness RF antenna fabrication, but also exhibited high wireless powering efficiency under mechanical deformation.^[^ ^106d^ ^]^ Simple and inexpensive large‐scale precise patterning method is required for mass production and commercialization of LM‐based RF electronic device. Therefore, noble patterning method for generating stable LM pattern onto dielectric surfaces should be further studied. Methods studied so far are pressure‐based filling and actuation methods,^[^ ^80^, ^83^, ^125^ ^]^ microfluidic channel wiring methods,^[^ ^79^, ^115^ ^]^ and electrowetting methods.^[^ ^82^, ^126^ ^]^

#### 4.2.2 Soft Robotics

Soft electronic devices can be integrated with soft actuators. The integrated system is called soft robotics, which is being widely studied for its applicability with wearable electronics and flexibility derived human‐friendly technology.^[^ ^103^, ^127^ ^]^ Main components for LM‐based soft robots include actuators,^[^ ^40^, ^80^, ^128^ ^]^ sensors,^[^ ^129^ ^]^ and computing circuit.^[^ ^128^, ^130^ ^]^ Especially, reconfigurability and restorability stemming from LMs fluidity provide advantages for manufacturing various kinds of damage‐free and modifiable soft robots.^[^ ^64^, ^131^ ^]^ At the same time, liquid metal‐embedded elastomer composites can also be utilized to provide high deformability and durability while maintaining stable electrical properties.^[^ ^129b^ ^]^ For example, Markvicka et al. fabricated restorable LM microdroplet‐embedded elastomer actuator and controlled its movement. The actuator required mechanical sintering to create conductive traces, which was reconfigured to maintain the originally intended circuit design when external damage was imposed.^[^ ^64d^ ^]^ Zhou et al. presented LM‐core flexible cable fabricated by coaxial‐printing of silicone rubber and Galinstan. The group integrated snake‐like soft robot with the cable and measured its motion and deformation in real‐time.^[^ ^42h^ ^]^ Patterning methods capable of stable integration with rigid components, large‐scale patterning, and short‐time fabrication should be developed for the popularization of LM‐based soft robotics.

#### 4.2.3 Heterogeneous Catalyst

Heterogeneous catalyst has been intensely researched because of its own advantages stemming from different phase between catalyst and reactants (or products) such as facile separation after catalysis, catalyst recyclability, and fast mass production.^[^ ^131^ ^]^ LM is considered as a promising candidate for heterogeneous catalyst because it provides large surface area for catalytic reaction site, serves as metal solvent, exhibits high selectivity, and consume slow energy.^[^ ^132^ ^]^ For example, Esrafilzadeh et al. demonstrated room temperature CO_2_ reduction to solid carbon species by liquid metal electrocatalyst mixed with metallic elemental cerium nanoparticles. The group utilized that cerium oxide catalyst forms at the interface of LM and electrolyte. This room temperature catalysis was realized because LM could dissolve metallic particles in ambient condition with enough concentration.^[^ ^133^ ^]^ Since catalyst's reaction property can be altered by modifying surface morphology or patterned structure,^[^ ^134^ ^]^ morphological or structural design of LM‐based catalyst can maximize the usage of LM as a heterogeneous catalyst.^[^ ^132^ ^]^ As an example of catalyst patterning, Paul et al. fabricated cathode catalyst layers with arrays of microcylindrical holes. It exhibited better performance in water management for proton exchange membrane fuel cells (PEMFCs) than continuous cathode catalyst layers. Since poor water management leads to flooding within the PEMFCs system, the patterned cathode catalyst layers had twice better mass activity than continuous one. When LMs outstanding catalytic properties are combined with advanced LM patterning techniques, it is expected that catalysis industry will develop much more than current state.

## Conflict of Interest

The authors declare no conflict of interest.
